# MicroRNA Microarray Profiling during Megakaryocyte Differentiation of Cord Blood CD133+ Hematopoietic Stem Cells

**DOI:** 10.22074/cellj.2018.5021

**Published:** 2018-03-18

**Authors:** Mohammad Houshmand, Mozhde Nakhlestani Hagh, Masoud Soleimani, Amir Ali Hamidieh, Saeed Abroun, Mahin Nikougoftar Zarif

**Affiliations:** 1 Blood Transfusion Research Center, High Institute for Research and Education in Transfusion Medicine, Tehran, Iran; 2 Department of Clinical and Biological Sciences, University of Turin, San Luigi Gonzaga Hospital, Orbassano, Italy; 3 Department of Hematology, Faculty of Medical Sciences, Tarbiat Modares University, Tehran, Iran; 4 Children’s Medical Center, Tehran University of Medical Science, Tehran, Iran; 5 HSCT Research Center, Shahid Beheshti University of Medical Sciences, Tehran, Iran

**Keywords:** Cord Blood, Hematopoietic Stem Cells, Megakryocytes, Microarray Analaysis, MicroRNAs

## Abstract

**Objective:**

In order to clarify the role of microRNAs (miRNA) in megakaryocyte differentiation, we ran a microRNA microarray experiment to measure the expression level of 961 human miRNA in megakaryocytes differentiated from human umbilical cord blood CD133+ cells.

**Materials and Methods:**

In this experimental study, human CD133+ hematopoietic stem cells were collected from three human umbilical cord blood (UCB) samples, and then differentiated to the megakaryocytic lineage and characterized by flow cytometry, CFU-assay and ploidy analysis. Subsequently, microarray analysis was undertaken followed by quantitative polymerase chain reaction (qPCR) to validate differentially expressed miRNA identified in the microarray analysis.

**Results:**

A total of 10 and 14 miRNAs were upregulated (e.g. miR-1246 and miR-148-a) and down-regulated (e.g. miR- 551b and miR-10a) respectively during megakaryocyte differentiation, all of which were confirmed by qPCR. Analysis of targets of these miRNA showed that the majority of targets are transcription factors involved in megakaryopoiesis.

**Conclusion:**

We conclude that miRNA play an important role in megakaryocyte differentiation and may be used as targets to change the rate of differentiation and further our understanding of the biology of megakaryocyte commitment.

## Introduction

Hematopoietic stem cells (HSC) are adult stem cells with self-renewal and multi-lineage differentiation properties, and thus able to produce diverse cell types such as megakaryocytes (1, 2). Meanwhile, the bone marrow microenvironment plays a pivotal role in regulating proliferation and differentiation of HSC (3). Also, there is a close contact between bone marrow niche and megakaryocytes where osteoblast cells, as the main component of osteoblastic niche, support megakaryopoiesis by releasing different growth factors (4). There are many factors such as cytokines, transcription factors and noncoding RNA that control the rate of HSC differentiation and proliferation (5). For instance, SCL, a transcription factor, enhances the development of megakaryocyte and erythroid lineages from hematopoietic stem cells (6). However, one of the most important factors implicated in differentiation and proliferation of stem cells is microRNA (miRNA) based regulation (7). 

MiRNA are endogenous non-coding RNA species that regulate gene expression and thus have an impact on differentiation, proliferation, apoptosis and other key biological processes (8, 9). Studies have shown that dysregulation of miRNA have an adverse effect on cell biology and sometimes lead to a disorder (10, 11). For example, in chronic myeloid leukemia (CML), miR-30e is expressed at low levels and up-regulation of this miRNA results in suppression of proliferation and apoptosis of k562 cells (12) or dysregulation of miR-126 enhances leukemogenesis (13).

Different miRNA determine cell fate decision of HSCs (14). Studies have shown that down-regulation of miR-10a results in differentiation of megakaryocytes from human umbilical cord blood CD133+ cells (15), while dysregulation of miR-486-3p induces erythroid differentiation from HSC and therefore restrain megakaryocyte differentiation (16). Moreover, another study has demonstrated that miR34a, mir146a, mir145 and mir150 enhance and mir150 inhibits megakaryocyte differentiation (17). Given the important regulatory role of miRNA in megakaryocyte differentiation and lack of sufficient data in this field, we ran a miRNA microarray experiment to examine the expression level of a large set of miRNA in megakaryocyte differentiation from human umbilical cord blood CD133+ cells. We provide new insights into the biology of megakaryopoiesis and megakaryocyte disorders. 

## Materials and Methods

In this experimental study, three human umbilical cord blood (UCB) samples were collected from donors who had a normal full-term vaginal delivery without any complications. All signed a written consent form according to the Iran Blood Transfusion Organization Ethics Committee standards. These samples were collected in cord blood bags (JMS, Korea) containing 22 ml Citrate Phosphate Dextrose Adenine.

### Separation of mononuclear cells

UCB samples were diluted 2:1 with phosphate buffer saline (PBS) and mononuclear cells were separated using Ficoll-Hypaque density centrifugation (density 1077 g/ cm3, Pharmacia, Sweden) at 2500 rpm for 30 minutes. The mononuclear cell layer was collected and washed with PBS containing 5% bovine serum albumin (BSA, Stem Cell Technology, Canada). The viability of cells was assessed by propidium iodide (PI) using flow cytometry.

### CD133+ cell isolation

The CD133^+^ cells were enriched by the magnetic activated cell sorting (MACS) method (Miltenyi Biotec, Canada) according to the manufacturer’s instructions. Repeating the procedure resulted in higher purity of the selected CD133^+^ cells. The efficiency of purification was verified by flow cytometry (Partec PAS III, Germany), with cells counterstained with the monoclonal antibodies (moAb) CD133-PE and CD34-FITC (Miltenyi, Canada) since most CD133+ cells also express CD34. In addition, moAb CD41-PE and CD61-FITC (DAKO, Denmark) were used to confirm the negativity of the megakaryocyte cells within separated cells.

### Cell culture, expansion and differentiation 

UCB CD133^+^ cells were then cultured in serum free stem span medium (Stem Cell Technology, Canada) in a tissue culture flask and incubated it at 37°C in a fully humidified atmosphere with 5% CO_2_. Stem cell factor (SCF, 100 ng/ml) and thrombopoietin (TPO, 100 ng/ ml) were added to the culture media for a week. Next, to achieve Mk differentiation, the cells were counted and transferred into 6-well tissue-culture plates with serum free stem span media containing TPO (100 ng/ml) for a week. The cytokines were replaced twice a week. Differentiation was followed by flow cytometry analysis of CD41 and CD61 surface marker expression. Colony forming unit-Mk (CFU-Mk) for colonogenic capacity and DNA analysis for ploidy detection of Mk progenitors were undertaken subsequently.

### Megakaryocyte characterization

#### Flow cytometry analysis

The cells were stained with FITC-conjugated anti-CD41 and PE-conjugated anti-CD61 (Dako, Denmark). Briefly, 1×10^6^ cells were incubated with 5µl of both moAb for 45 minutes at 4°C. The isotype control antibodies were used as negative controls. Prior to analysis, the incubated cells were washed twice with PBS containing 1% BSA. 

#### Colony forming unit-Mk

To evaluate the colonogenic capacity of Mk differentiated cells, we used MegaCult (Stem Cell Technologies, Canada), a medium that is formulated to allow optimal detection of Mk progenitors. One thousand differentiated cells were added to 2.0 ml of MegaCult and 1.2 ml of cold collagen solution, and after mixing, were then transferred into two 35 mm Petri dishes, which were placed in a 100 mm Petri dish along with another 35 mm Petri dish containing 3 ml sterile water to maintain optimal humidity. After 14 days, the colonies in each Petri dish were counted.

#### Ploidy analysis 

The ploidy of Mk differentiated cells was assessed by flow cytometry using DNA binding to PI. For this purpose, the cells were incubated for 45 minutes at 37°C with 0.1% Triton X-100 (Sigma, USA), 25 mg/ml RNAse (Sigma, USA) and 10 mg/ml PI (Sigma, USA). 

### Isolation of Mk differentiated cells by MACS for microarray analysis 

For maximum purity, Mk differentiated cells were isolated by MACS using bead-conjugated CD61 moAb (Miltenyi Biotec, Canada) according to the manufacturer’s instructions. The efficiency of purification was verified by flow cytometry, with cells counterstained with CD41FITC and CD61-PE. 

### MicroRNA microarray analysis 

The miRNA microarray experiment was run using the Agilent Human miRNA microarray platform (Agilent Technologies, USA) at the Centre for Genomic Regulation (CRG, Barcelona, Spain). This microarray chip contains probes for 961 human miRNAs from the www.miRbase. org. Total RNAwas extracted from MACS-sorted CD133+ and Mk differentiated cells using Trizol (Invitrogen, USA). RNA quality control, labeling and hybridization were performed at CRG according to the protocols of the Agilent miRNA microarray system. Microarray slides were scanned with the Agilent Microarray Scanner. Feature Extraction software (version 10.7) was used to convert the microarray image information into spot intensity values(Agilent Technologies, USA). The signal intensities, after background subtraction, were imported directly into GeneSpring 11.0 (Agilent Technologies, USA) for quintile normalization prior to statistical analyses. 

### Quantitative polymerase chain reaction 

Microarray results were validated for a number of dysregulated miRNAs by quantitative polymerase chain reaction (qPCR). MiRNAs was extracted from CD133+ and differentiated megakaryocyte cells using Trizol (Qiagen, Hilden, Germany). We used the miRNA qRT-PCR detection kit (Stratagene, Houston, TX, USA) containing reagents that are sufficiently sensitive for cDNA synthesis and miRNA amplification. The universal reverse primer, downstream primer, anneals to the universal tag and is added to the cDNA sequence in reverse transcription reaction. The forward primer was designed according to the sequence and length of a miRNA, providing specificity of the qPCR reaction. The miRNA qPCR master mix contained the EvaGreen® dye, which is more stable than its similar counterpart SYBR® Green I dye. MiRNA expression levels were quantified by the Rotor-Gene 6000 system (Corbett, Auckland, New Zealand). Relative expression was calculated using the ..CT method (18) with the endogenous U6 snRNA as the internal control.

### Integration between miRNA and mRNA 

To identify regulated megakaryocytic differentiation- associated mRNAs, the targets of differentially expressed miRNA were obtained from a web-based prediction tool, namely the human miRBase Targets (http://micro-rna. sanger.uk/targets/v3/).

### Statistical analysis

Statistical analysis of microarray data was undertaken using the Bioconductor limma package (http:// bioconductor.org/packages/2.5/bioc/html/limma.html). Results of qPCR were analyzed by ANOVA and data are presented as means ± SD. P<0.05 was considered significant. 

## Results

### CD133+ cell isolation 

**Fig.1:**
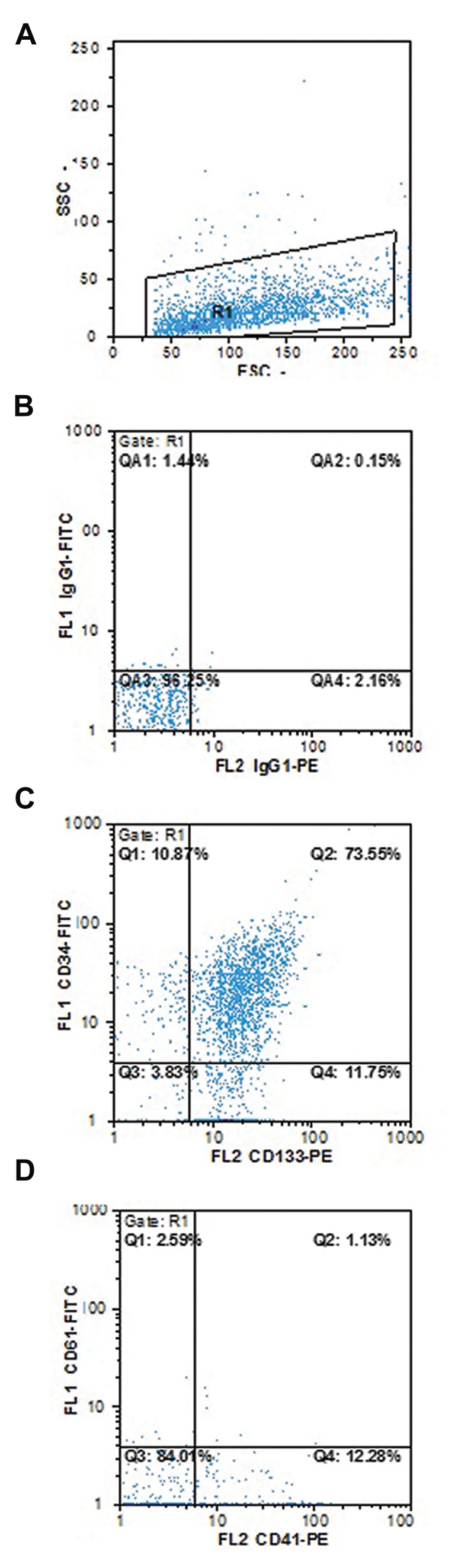
Purity of isolated CD133+ cells. A. Cell distribution, B. Control isotype, C. CD133+ versus CD34+ cells distribution, and D. CD41+ versus CD61+ distribution.

The CD133+ cells were separated by MACS. The percentage of isolated CD133+ cells in the three UCB samples was 89.5 ± 10.9 (Fig .1). 

### Megakaryocyte differentiation 

**Fig.2:**
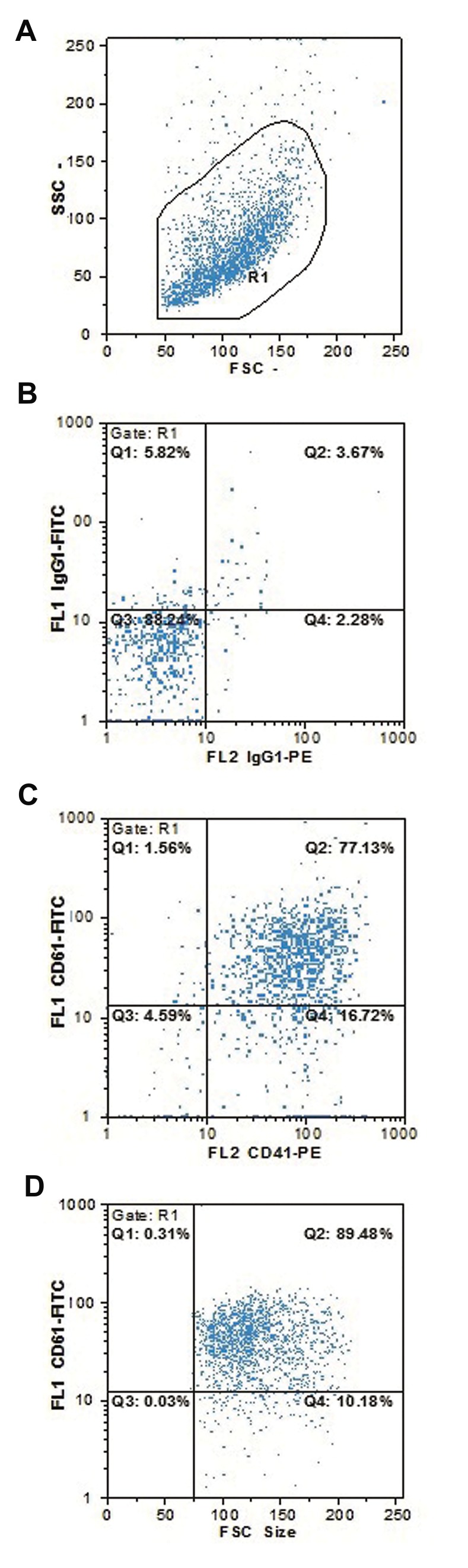
Megakaryocytic differentiated cells. A. Cell distribution, B. Control isotype, C. CD41+ versus CD61+ cells distribution, and D. The purity of CD61+ cells after MACS sorting.

According to flow cytometry analysis, 79.5 ± 16.9% of cells were differentiated into the megakaryocyte series and expressed both CD41 and CD61 (Fig .2A-C). In order to maximize the purity of megakaryocyte differentiated cells, we used bead-conjugated CD61 moAb and the MACS method. After purification, 92.3 ± 5.9% of cells were positive for CD61 (Fig .2D).

### CFU-Mk assay

**Fig.3:**
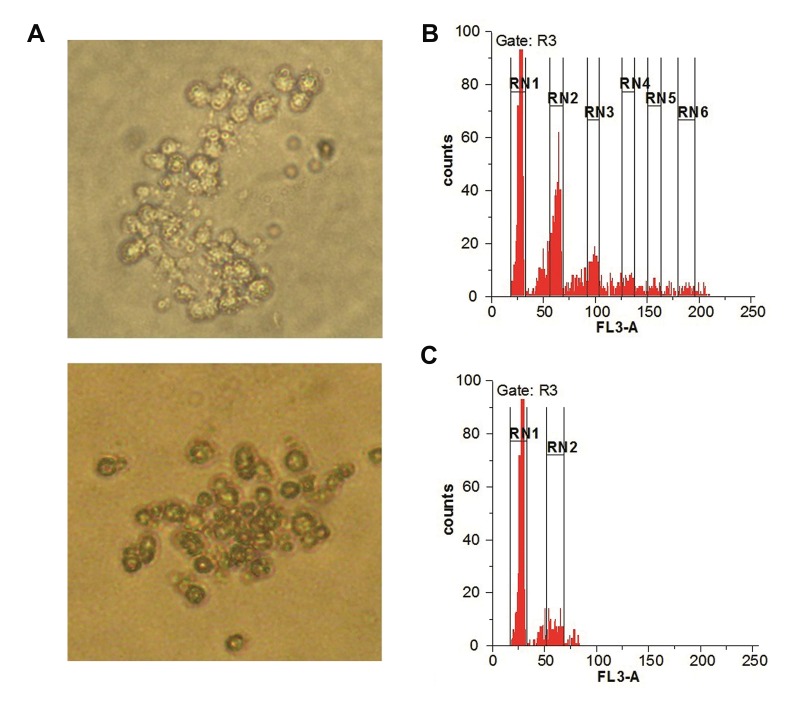
Colony forming and DNA analysis of differentiated cells. **A.** The colonies formed from megakaryocytic differentiated cells in megacult medium (×400), **B.** Ploidy analysis of megakaryocytic differentiated cells, RN1 to RN6 represent G0G1 peaks of 2N, 4N, 8N and 16N populationsrespectively, and **C.** Ploidy analysis in CD133+ hematopoietic stem cells, RN1 represents G0G1 peaks of 2N population.

The CFU assay was undertaken to evaluate the colongenic capacity of megakaryocytic differentiated cells. The Mk colonies formed in the MegaCult medium showed variable sizes with 43.2 ± 19.5 small size colonies containing 3-20 cells, 6.6 ± 3.5 medium size colonies containing 20-49 cells and 2.3 ± 1.5 large size colonies containing at least 49 cells (Fig .3A).

### Ploidy analysis 

DNA analysis of differentiated cells showed the percentage of 38 ± 7.2, 31 ± 8.5, 14 ± 5.6 and 7.2 ± 3.0 in 2N, 4N, 8N and 16N populations respectively (Fig .3B, C). In addition, some cells with higher ploidy were observed. However, more than 90 percent of CD133+ cells were in the diploid cycle. 

### miRNA expression profile in CD133+ stem cells and megakaryocytes

**Fig.4:**
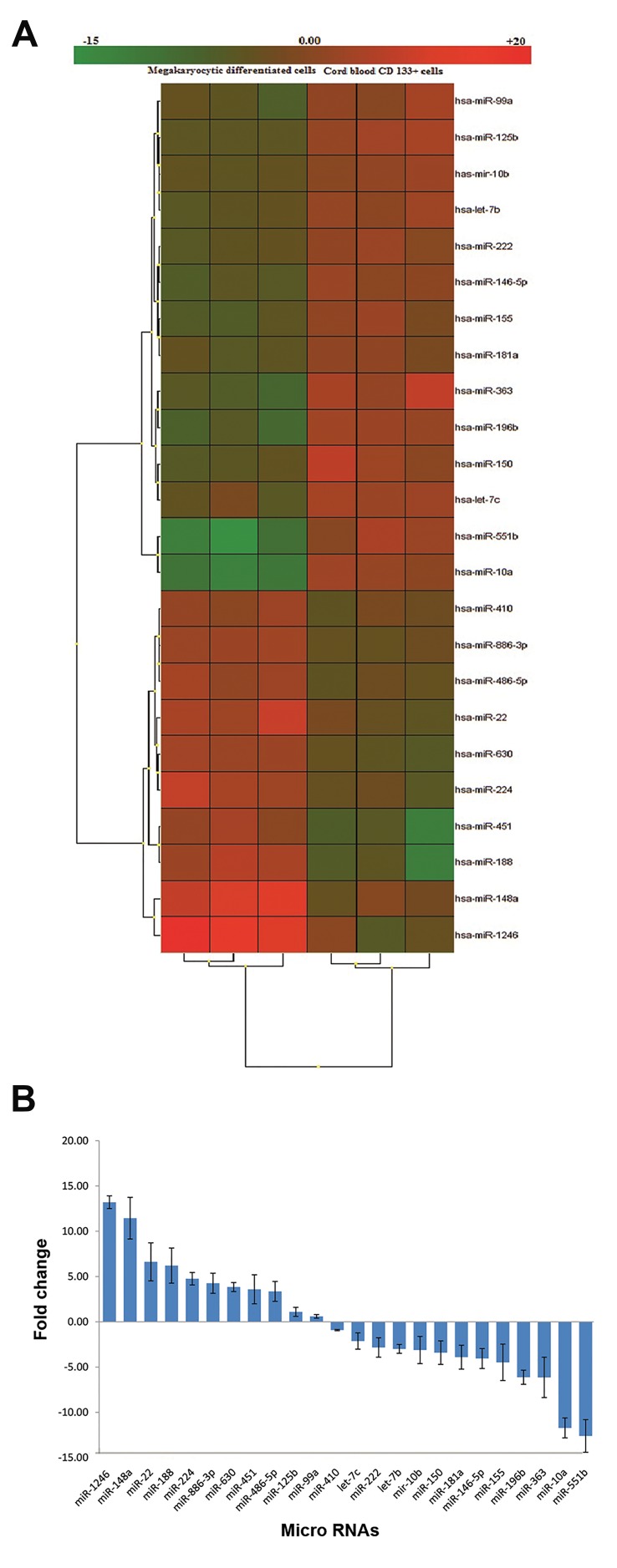
Alteration of miRNAs during megakaryocytic differentiation ofCD133+ hematopoietic stem cells. A. Hierarchical clustering of 24 miRNAs by microarray analysis and B. Fold change expression of miRNAs by realtime polymerase chain reaction (PCR).

The expression profile obtained from the miRNAmicroarrayshowed a total of 24 differentially expressed miRNAbetweendifferentiated megakaryocytes and undifferentiated CD133+ cells (Fig .4A). Among these, ten were significantly up- regulated in megakaryocytes with the top four being miR1246, miR-148a, miR-22 and miR-188 from 18 to 5 fold increase. The other 14 miRNA showed significant down- regulation with miR-551b, miR-10a, miR-363 and miR-196b from 12 to 6 fold decrease displayed the highest fold-change. 

### MiRNA quantitative polymerase chain reaction

To validate microarray-based differentially expressed miRNAs, the expression level of twenty four miRNAs with highest fold changes were examined using qPCR. All these miRNAs were confirmed as differentially expressed (Fig .4B).

### Integration between miRNAs and mRNA 

 We gathered the targets of the dysregulated miRNA by using the prediction resources MiRanda and ComiR. The main mRNA-coding targets changed during megakaryocyte differentiation are summarized in Table 1. For instance, classical dendritic cells (*CDC*), stem cell leukemia (*SCL*) and B Cell Lymphoma-2 (*BCL-2*) are the main targets for down-regulated miRNA (Table 1). On the other hand, the main targets of down-regulated miRNAs were Runt-related transcription factor 1 (*RUNX-1*), C-X-C chemokine receptor type 4 (*CXCR-4*) and Myeloblastosis (*MYB*) (Table 2).

**Table 1:** Associated genes with up-regulated miRNAs in megakaryocytopoiesis


	*DYRK1A*	*MAPK*	*CDC*	*SCL*	*BCL-2*	*IGF-1*	*KLF*	*CXCR4*	*MAX*	*WNT*	*NFAT*

miR-22		⬛	⬛						⬛		
miR-148a	⬛		⬛	⬛	⬛	⬛					⬛
miR-188		⬛	⬛				⬛				
miR-224			⬛					⬛			
miR-410				⬛	⬛	⬛			⬛	⬛	
miR-451							⬛				
miR-486-5p			⬛	⬛	⬛	⬛					
miR-630				⬛	⬛						⬛
miR-886			⬛								
miR-1246	⬛	⬛									


**Table 2:** Associated genes with down-regulated miRNAs in megakaryocytopoiesis


	*CDK*	*MYC*	*AGTR-2*	*HOX*	*RUNX-1*	*JUN*	*CXCR4*	*PPARA*	*MYB*	*c-Myb*	*ETS*	*LIN28*	*CREBBP*

miR-Let7b			⬛		⬛	⬛							
miR-Let7c	⬛	⬛											
miR-10a				⬛	⬛	⬛	⬛						
miR-10b				⬛			⬛	⬛					
miR-125b					⬛								
miR-99a					⬛								
miR-146b-5p	⬛						⬛		⬛				⬛
miR-150						⬛		⬛	⬛				
mir-155					⬛	⬛		⬛	⬛		⬛		
miR-181a			⬛		⬛				⬛			⬛	⬛
miR-196b				⬛									
miR-363	⬛												
miR-551b	⬛												


## Discussion

Thrombocytopenia usually occurs following bone marrow failure and leads to life-threatening hemorrhages. Increase in demand for platelet transfusion and platelet transfusion refractoriness in multi-transfused patients has resulted in basic and clinical studies focusing on platelet production from HSCs. *In vitro* production of platelets essentially relies more on the biology of megakaryopoiesis and the signals involved in this pathway (19).

Megakaryocytes develop from HSCs through multiple subsequential commitment steps, namely common megakaryocyte and erythroid progenitor formation, megakaryocyte differentiation, surface marker acquisition, nuclear polyploidization and cytoplasmic maturation (20, 21). Multiple environmental and molecular mechanisms control the fate of HSC. Transcription factors play a key role in differentiation and maturation development. For instance, RUNX-1, Gata binding protein 1 (GATA1), Friend leukemia integration 1 (FLI-1), T-cell acute lymphocytic leukemia protein 1 (TAL-1) play important roles in Mk lineage commitment (19, 22). 

MiRNA, regulate post-transcriptional gene expression by targeting mRNAs and thus inhibit translation. Therefore, to ascertain the role of these small molecules in megakaryopoiesis, the expression profile of miRNAs in CD133+ and Mk derived CD133+ cells was analyzed. To ensure Mk differentiation, the cells were evaluated morphologically and functionally. The presence of polyploid cells confirmed normal Mk formation. Furthermore, colony formation capacity of differentiated cells in the MegaCult media confirmed the functional property of cells. 

The qPCR-based expression analysis of the top six dysregulated genes identified in the microarray experiment showed high consistency across the two methods. Opalinska et al. (23) demonstrated that 13 and 81 miRNA were up-regulated and down-regulated respectively among a total of 435 miRNA investigated in Mk differentiation of murine hematopoietic progenitor cells. The expression pattern of a number of miRNA was similar to human megakaryopoiesis, most likely due to the close human and murine hematopoiesis process. 

Differentiation of Mk from human CD34+ HSCs in Garzon et al. (24) also showed dysregulation of 28 miRNAs, of which the expression pattern of 16 was consistent with the present study. Immaturity of CD133+ HSCs in comparison to CD34+ HSCs may justify the overall differences between miRNA expression patterns in these studies. Overall, different patterns in several studies may also be due to variation in the sources of HSCs, differentiation induction methods and the stage of differentiation.

Given that miRNA control biological processes via targeting mRNAs, the study of these targets may shed light on their role in this pathway. Some altered miRNA have common targets that confirm the combinatorial model of gene expression control. For instance, miR10a, let-7b, miR-181b, miR-125a, miR-99a and miR-155 target the RUNX-1 transcription factor (25). Deletion of *RUNX-1* leads to rapid and prolonged drop in platelet counts in adult mouse (22). Therefore, 12-, 3- and 4-fold decrease in miR-10a, let-7b and miR-155 may induce overexpression of *RUNX-1* as their main target.

Romania et al. (26) showed that miR-155, expressed in hematopoietic progenitor cells, is sharply down- regulated during Mk differentiation and thus resulted in the overexpression of ETS Proto-Oncogene 1 (*Ets-1*) and Myeloid Ecotropic Viral Integration Site 1 (*Meis-1*). These transcription factors have well-known functions in the Mk series. In addition, miR-155 targets *RUNX-1, JUN, Peroxisome proliferator-activated receptor alpha gene (PPARA), MYB* and *Ets-1*, all of which are involved in this pathway (27). These transcription factors have key roles in megakaryocytic-erythroid progenitor cell fate, and alteration of these molecules, especially MYB, may alter the lineage differentiation fate decision between MK and Erythroid series (16). MiR-10a has putative targets in the HOX transcription factor family with the highest sequence compatibility with *HOXA1* mRNA. Direct interaction of miR-10a and the 3´UTR of *HOXA1* mRNA was demonstrated by a luciferase reporter assay both *in vitro* and *in vivo* by Garzon et al. (24). The *HOX* gene family has an important role in the proliferation and lineage differentiation of HSC with *HOXA1* expression up-regulated at the transcript and protein levels during Mk differentiation. Thus, down-regulation of miR10a may control Mk differentiation via *HOXA1* posttranscriptional suppression.

The *c-MYB, CDK* and *LIN28* also play important roles in MK commitment. Given the expression of miR181, miR146b-5p, miR-150 and miR-155 was down- regulated during megakaryopoiesis and their sequence complementarity to mRNA of these three key genes, it is possible that these genes are overexpressed during Mk formation (28, 29). Stem cell properties including self- renewal, quiescence and capacity to overcome senescence have all been shown to be under the control of certain miRNA (30).

During the differentiation process, downregulation of some miRNA are reported. For instance, miR-551b inhibits hematopoietic stem cell differentiation and its down-regulation is crucial for hematopoiesis. In this study, miR-551 showed significantly reduced expression through megakaryopoiesis (12.32-fold). In agreement with Petriv et al. (31), we show miR-148 up-regulation in megakaryocytes. MiR-148 targets *CDC, SCL* and *BCL-2*, and may decrease the self-renewal property of progenitor cells and apoptosis, leading to the differentiation process (32). 

Another overexpressed miRNA was miR-451. Zhang et al. (33) demonstrated that miR-451 is required for late maturation of the erythroid series. Many miRNA were overexpressed during erythropoiesis which were also up- regulated during megakaryocytic differentiation; these miRNAs facilitate a number of cellular processes that are common in both lineages. According to Polioudakis et al. (34), overexpression of miR-22 helps to terminate hematopoietic differentiation through targeting the Max protein to inhibit the Myc-Max transcriptional complex.

Consistently, miR-22 showed increase in expression during Mk differentiation. The highest expression change was observed for miR-1246 which targets the transcripts of mitogen-activated protein kinases (*MAPK*) and dual- specificity tyrosine phosphorylation-regulated kinase 1 (*DYRK-1A*) and cell adhesion molecule 1 (*CADM1*). There is, however, no evidence of its role in megakaryopoiesis. Study of the miR-1246 function showed many zinc finger protein targets for this molecule. There are some other targets that showed no obvious correlation with this lineage, however, miR-1246 has recently been used as a diagnostic and prognostic biomarker in a number of cancers such as oesophagous squamous cell carcinoma and hepatocellular carcinoma (35, 36), thus requiring further investigation. Overall, our data suggest that miRNA play a crucial role in megakaryocyte differentiation by providing new insight into the molecular mechanism of hematopoiesis. 

## Conclusion

The diverse expression changes of miRNA during megakaryopoiesis and their targets, which are almost all transcription factors involved in megakaryocytic differentiation, reveals an important role of these molecules in platelet biogenesis. Further investigations may lead to a more detailed molecular mechanism of megakaryopoiesis.
